# Supplementary material for: Adherence to Guideline-Directed Medical Therapy in Hospitalized Older People with Heart Failure at Discharge and 3-Month Follow-Up
Source: J Clin Med. 2025 Sep 30;14(19):6928. doi: 10.3390/jcm14196928 (PMC12525149; doi:10.3390/jcm14196928)
Supplement: Supplementary file 1 [file jcm-14-06928-s001.zip › jcm-3865912-supplementary.pdf]

Supplementary documents

Table S1: Recommended target dose.

Medication	Recommended target dose
Lisinopril	20-35mg o.d.
Enalapril	10-20mg b.i.d.
Captopril	50mg t.i.d.
Perinodopril	4mg o.d.
Sacubitril/valsartan	97/103mg b.i.d.
Candesartan	32mg o.d.
Losartan	150mg o.d.
Valsartan	160mg b.i.d.
Telmisartan	80mg o.d.
Irbesartan	300mg o.d.
Metoprolol	200mg o.d.
Carvedilol	25mg b.i.d.
Bisoprolol	10mg o.d.
Atenolol	100mg o.d.
Spironolactone	50mg o.d.
Eplerenone	50mg o.d.
Empagliflozine	10 mg o.d.
Dapagliflozine	10 mg o.d.

Supplementary figure 1

S1A: GDMT use at discharge vs FU

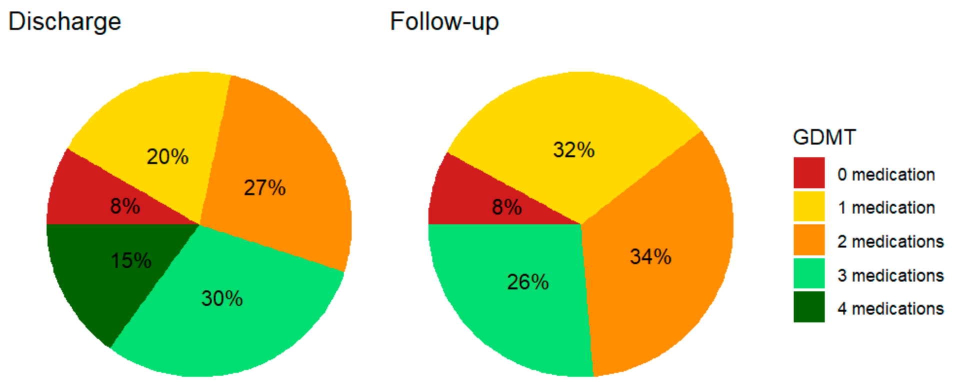

S1B: GDMT use at discharge vs FU according to admission specialty

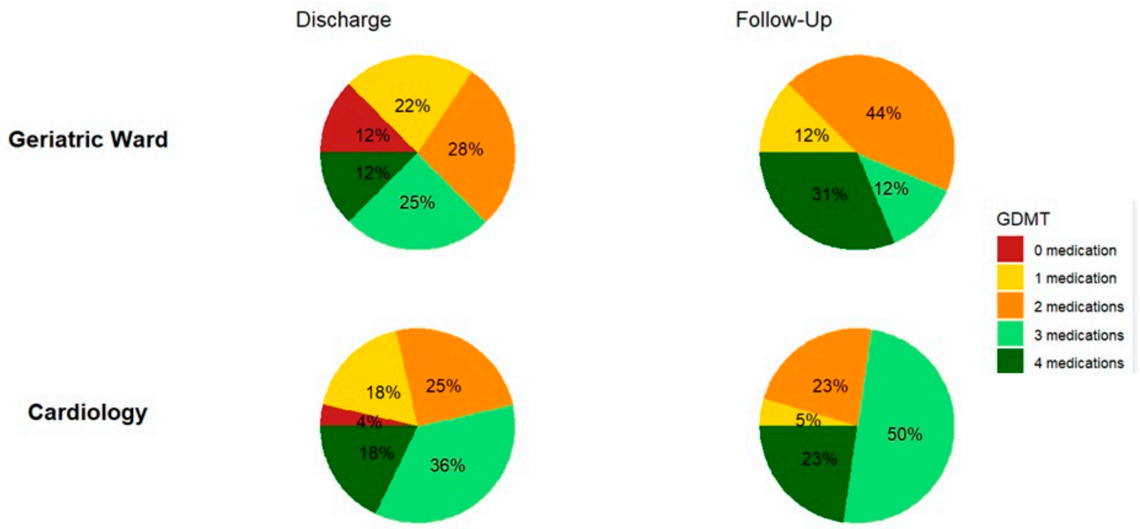

S1C: GDMT use at discharge vs FU according to age group

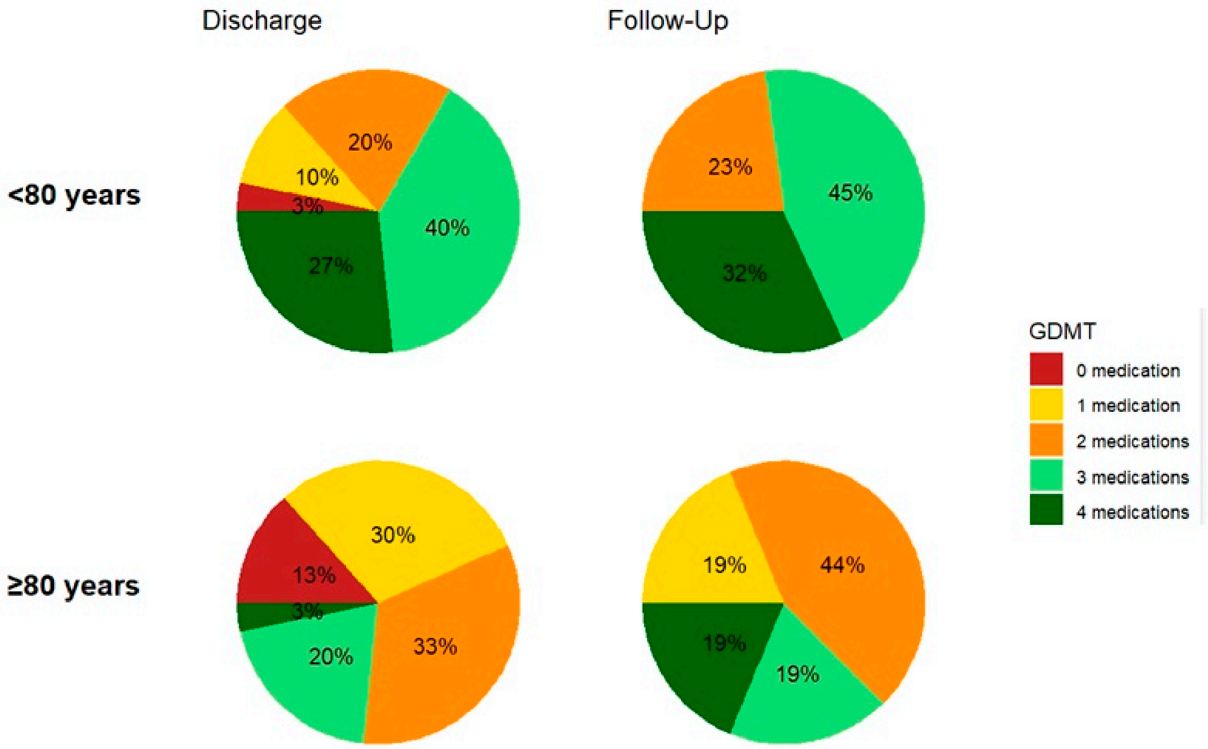

S1D: GDMT use at discharge vs FU for other determinants

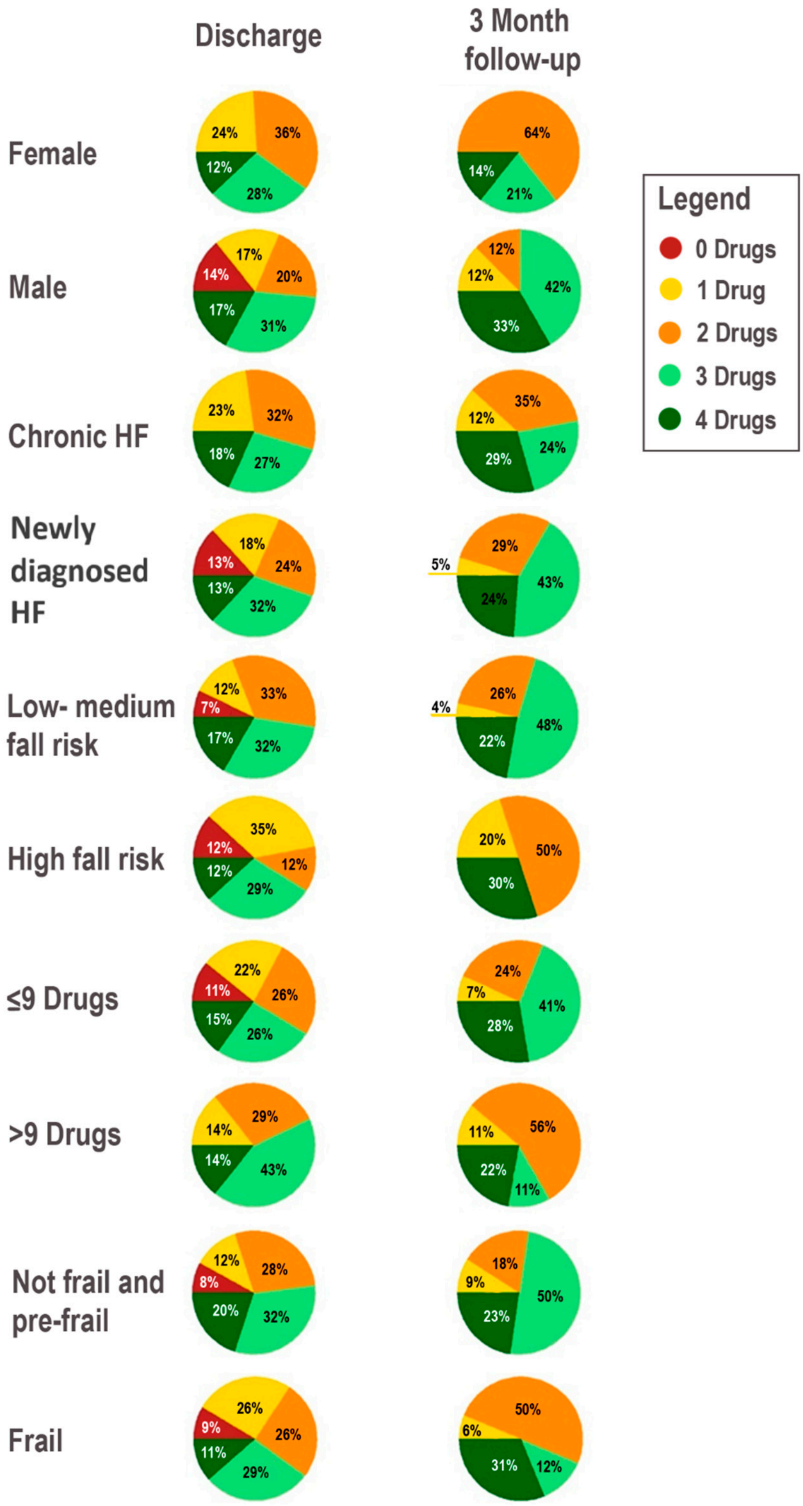

Figure S1 - GDMT use based on sex (p-value at discharge 0.72, at FU 0.33), type of HF (p-value 0.71, 0.85), fall risk (p-value 0.72, 0.72), polypharmacy (p-value 1.00, 1.00) and frailty (p-value 0.47, 0.83) on discharge and three months
